# Electroacupuncture in the treatment of IBS in rats: investigation of the mechanisms of CRH^+^ neurons in the paraventricular nucleus

**DOI:** 10.1152/jn.00156.2023

**Published:** 2023-07-12

**Authors:** Fang Gao, Wei-Hua Yuan, Sheng-Bing Wu, Zi-Bei Wang, Guo-Qi Zhu, Mei-Qi Zhou

**Affiliations:** ^1^Key Laboratory of Xin’an Medicine, the Ministry of Education and Key Laboratory of Molecular Biology (Brain diseases), Anhui University of Chinese Medicine, Hefei, China; ^2^Bengbu Medical College, Bengbu, China

**Keywords:** corticotropin-releasing hormone, corticotropin-releasing hormone receptor 1, corticotropin-releasing hormone receptor 2, electroacupuncture, irritable bowel syndrome

## Abstract

Electroacupuncture (EA) is well documented to treat irritable bowel syndrome (IBS). However, the mechanism of the central nervous system related to IBS and acupuncture stimulation is still not well known. In this study, a rat model of IBS was established by cold-restraint comprehensive stresses for 15 days, and it was found that the levels of corticotropin-releasing hormone (CRH), corticosterone (CORT), and adrenocorticotropic hormone (ACTH) in the peripheral serum were increased; the visceral sensitivity was enhanced; and the intestinal motility was accelerated, specifically, there was an enhancement in the discharge frequency of neurons in the paraventricular nucleus (PVN). EA treatment for 3 days, 20 min/day, alleviated the increase in the levels of CRH, CORT, and ACTH in the peripheral serum of rats, reduced the visceral sensitivity of IBS rats, and inhibited colon movement and discharge frequency of the neurons in the PVN. In addition, EA could reduce the excitability of CRH neurons and the expression of corticotropin-releasing hormone receptor 1 (CRHR1) and corticotropin-releasing hormone receptor 2 (CRHR2) in PVN. At the same time, the expression of CRH, CRHR1, and CRHR2 in the peripheral colon was decreased. Taken together, EA appears to regulate intestinal functional activity through the central CRH nervous system, revealing the central regulation mechanism of EA in IBS rats, and providing a scientific research basis for the correlation among the meridians, viscera, and brain.

**NEW & NOTEWORTHY** The purpose of this research was to determine the central regulatory mechanism of electroacupuncture (EA) in rats with irritable bowel syndrome (IBS). Our results showed that combined with the serum changes in corticotropin-releasing hormone (CRH), corticosterone (CORT), and adrenocorticotropic hormone (ACTH), the improvement of IBS by EA was related to them. Furthermore, EA could regulate intestinal functional activity through the central CRH^+^ nervous system.

## Graphical abstract

**Figure:**
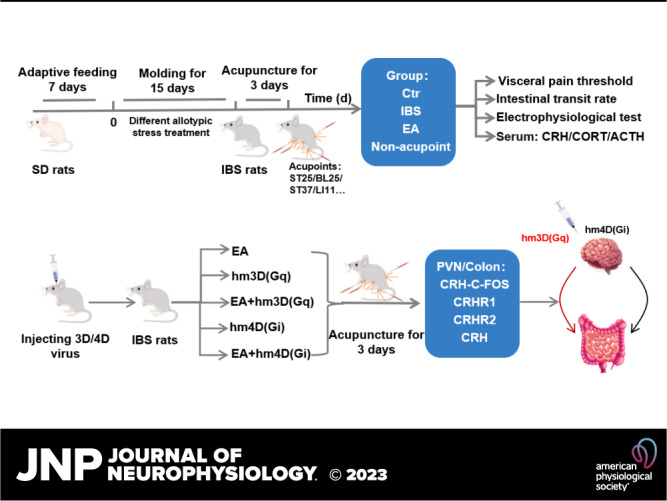

## INTRODUCTION

Irritable bowel syndrome (IBS) is a hereditary disease that affects people worldwide and has gained epidemic status (1). IBS was defined as a common nonorganic gastrointestinal dysfunction according to the Rome IV diagnostic criteria, with abdominal pain as the main clinical manifestation, accompanied by changes in the frequency or characteristics of defecation (2, 3). In Europe and North America, IBS primarily affects adults, especially women, and generally presents between the ages of 15 and 65 yr (4). In surveys and research from other countries, including China, 20%–29% of school-age children suffered from functional bowel disease, and up to 30% of infants aged <12 mo suffered from the disease (5), which results in huge economic pressure and burden to society and families (6). Recent studies have shown that IBS-induced abnormal gastrointestinal motility and visceral sensitivity are associated with paraventricular nucleus (PVN) and enteric nervous system dysregulation (7–9). However, to our best knowledge, there is no effective treatment for IBS.

Corticotropin-releasing hormone (CRH) is a key regulatory peptide that mediates the hypothalamus-autonomic nervous system pathway to various stress responses and plays an important role in stress responses. Changes in gastrointestinal motility are reportedly mainly related to the central effect of CRH (10). Mönnikes et al. (11) found that the PVN might be the neuroanatomical site of CRH-mediated gastrointestinal motility changes, and CRH was abundantly expressed in PVN (12). Studies have confirmed that CRH in the central nucleus affects the motor function of the peripheral gastrointestinal tract through the autonomic nervous pathway (13–15). It was also clearly shown that CRH was upregulated when the PVN was stressed, which in turn accelerated colonic transport through CRH receptor 1 (CRHR1) and inhibited gastric emptying through CRH receptor 2 (CRHR2) (16, 17). Furthermore, CRH signals in the PVN play an important role in the development and maintenance of stress-induced visceral hypersensitivity (18–20).

Acupuncture is an important part of traditional Chinese medicine, as an alternative therapy that can prevent and treat a variety of diseases, and it is increasingly recognized by people worldwide. Clinical studies have confirmed that acupuncture is effective in the treatment of IBS (21, 22), specifically, acupuncture could improve the IBS quality of life (IBS-QOL) score and reduce the IBS symptom severity score (IBS-SSS) (23, 24). At present, experimental studies have shown that acupuncture affects the function of the amygdala, raphe greater nucleus, periaqueductal gray (PAG), and PVN in relation to gastrointestinal disease (25–30). However, at present, there is a lack of systematic, comprehensive, and in-depth research about the mechanism of PVN’s participation in improving IBS symptoms by acupuncture. This study furthered previous research evidence (31).

Therefore, in this study, we determined the central nervous regulation mechanism of acupuncture intervention on intestinal functional activity in a rat model of IBS, and further explored the role and mechanism of the central CRH nervous system on intestinal functional activity in IBS rats with respect to acupuncture intervention, to analyze whether acupuncture regulates intestinal functional activity through the central CRH nervous system and provide a scientific basis for the relationship among the meridians, viscera, and brain.

## MATERIALS AND METHODS

### Animals

In total, 110 healthy male Sprague-Dawley rats of specific pathogen-free (SPF) grade (weight: 180 ± 20 g, 6-wk old) were purchased from the Laboratory Animal Center of Anhui University of Traditional Chinese Medicine, China. The license number was SYXK (Anhui) 2020-001. The rats were housed in a cage with an independent air supply system (Kangwei IR60) and exposed to a 12-h light/dark cycle at 20°C room temperature, and 55% humidity. All rats had ad libitum access to food and water. Body weight was recorded on alternate days. All experimental procedures were in strict compliance with the Guidelines on the Treatment of Laboratory Animals issued by the Ministry of Science and Technology, PRC in 2006, and were approved by the Animal Ethics Committee of Anhui University of Chinese Medicine, China (Approval Number: 2023012).

### Groups and Treatments

According to previous studies (32–34), two kinds of chronic unpredictable stress such as restraint, forced ice-water swimming, and a combination of ice-water mixture gavage with hunger and satiety were randomly selected every day to establish an animal model of chronic combined stress with multifactor interaction. After stimulation on the 15th day, the success of the IBS model was evaluated based on the following criteria: the rats had loose stools, weak limbs, slow movement, and sluggish reaction; body hair lost its normal luster and became sparse; and the rats showed increased visceral sensitivity. In the first part of the study, except for the control group (*n* = 15), the rats with IBS were randomly divided into three groups: IBS group (*n* = 15), electroacupuncture (EA) group (*n* = 15), and nonacupoint group (*n* = 15). In the second part of the study, rats were injected with hm4D(Gi) virus and hm3D(Gq) virus, respectively, in the PVN of the brain, and then the IBS model was replicated. Rats with IBS were randomly divided into five groups: hm4D(Gi) group (*n* = 10), EA + hm4D(Gi) group (*n* = 10), hm3D(Gq) group (*n* = 10), EA + hm3D(Gq) group (*n* = 10), and EA group (*n* = 10).

Rats in the EA, EA + hm4D(Gi), and EA + hm3D(Gq) groups were vertically acupunctured with stainless steel needles (diameter: 0.19 mm, needle length: 10 mm, Suzhou Acupuncture & Moxibustion Appliance Co., Ltd.) at ST25 (5 mm on the lateral side of the umbilical side), BL25 (located at the lower edge of the fourth lumbar spine process, 5 mm on the lateral side of the posterior midline), ST37 (located ∼5 mm below the back leg ST36), LI11 (located in the depression on the lateral front of the elbow joint), the depth of which was 4–5 mm, and then the needle was inserted into the pulse electrotherapy instrument (Suzhou Medical Appliance Factory Co., Ltd.). The frequency was 2 Hz, and the intensity of the dilatational wave was 1 mA. This treatment was carried out for 3 days, once a day, 20 min each time. The nonacupoint group rats were vertically acupunctured with stainless steel needles 2 mm outside the aforementioned acupoints. All the aforementioned operations were performed under isoflurane (RWD Life Science Co., Ltd., shenzhen) anesthesia.

### Determination of Visceral Pain Threshold and Intestinal Propulsion Rate

Visceral pain threshold determination was performed after the last EA in the first part of the study as follows: the rats were fasted for 12 h, and each rat was anesthetized under inhaled isoflurane. The rats were fixed on a transparent rat frame, and one end of the 8-Fr catheter with balloon was connected to a 5-mL syringe containing warm water, and the balloon was inserted at a distance of 4–5 cm from the anus of the rat. After the rats were fully awake, warm water was slowly injected into the balloon to gradually increase the pressure in the balloon, the injection volume threshold during abdominal elevation was recorded, and the average value was obtained after three repetitions carried out at 15-min intervals. For determination of intestinal propulsion rate, the rats were fasted for 24 h and given an oral dose of 10% activated carbon suspension for 20 min (0.8 mL/rat), and then euthanized by cervical dislocation under pentobarbital sodium anesthesia (Beijing Chemical Reagent Co., Ltd.). The intestinal propulsive rate was defined as follows: intestinal propulsive rate = the distance from the pylorus to the leading edge of the activated carbon suspension in the intestine/full length from the pylorus to the ileocecal region × 100%.

### CRH, ACTH, and CORT Assay

Following the determination of the visceral pain threshold, a blood sample from the abdominal aorta was collected while the intestinal propulsion rate was measured. The serum was separated by centrifugation after 24 h of refrigeration at 4°C. The concentrations of CRH, ACTH, and CORT in rat sera were detected by ELISA; the kits for CRH, ACTH, and CORT (E-EL-R0270c; E-EL-R0048c; E-EL-0160c.) were purchased from Elabscience Biotechnology Co., Ltd., and the quantification assays were carried out according to the manufacturer’s protocol.

### Multichannel In Vivo Recording Technology

Following the last EA in the first experiment, the neuronal discharge signals of control group rats, IBS group rats, nonacupoint group rats, and EA group rats were collected, and inhalation isoflurane anesthesia was performed by the small animal anesthesia machine. Then, the head of the rats was fixed by the stereoscopic brain locator. According to Paxinos and Watson’s rat head map, PVN regional coordinates (bregma: −1.8 mm, LR (L, left; R, right): 0.4 mm lateral to the midline, and *H*: 7.5–8.2 mm deep from the brain surface) were used. An eight-channel microfilament electrode was implanted in the craniectomy, and the tip of the electrode was slowly pushed into the PVN area with a manual micro-thruster. After the neuronal discharge was stable, the OmmiPlex multichannel acquisition system (Plexon Inc., Dallas, TX) was used to record the neuronal discharge and field potential (FP) for 5 min. Then, the PL2 files of neuronal firing and FP were obtained by filtering analysis using Neuro Explorer software of multichannel in vivo signal acquisition and processing system (v.5.029, Nex Technologies, Colorado Springs, CO). Finally, Offline Sorter (v.4.5.0, Plexon Inc.) was used to analyze the PL2 files.

### Chemogenetics

After 5 days of adaptation in the second experiment, 500 nL mixture of rAAV-CRH-Cre-WPRE-hGH-pA (PT-0588) and rAAV-Ef1α-DIO-hM3D(Gq)-mCherry-WPREs virus (PT-0042) [PT-0588, PT-0042, Brain VTA (Wuhan) Co., Ltd., China] was injected into the bilateral PVN region (bregma: −1.8 mm, LR: 0.4 mm, and *H*: 8 mm), under isoflurane anesthesia, to excite the CRH neurons. Similarly, 500 nL mixture of rAAV-CRH-Cre-WPRE-hGH-pA and rAAV-Ef1α-DIO-hM4D(Gi)-mCherry-Wpres-pA [PT-0043, Brain VTA (Wuhan) Co., Ltd., China] was injected into the bilateral PVN region to inhibit the CRH neurons. After 15 days of model replication, the rats were intraperitoneally injected with clozapine *N*-oxide (CNO) [Brain VTA (Wuhan) Co., Ltd., China] (3 mg/kg, 0.33 mg/mL). After 1 h, 20 min of EA was performed, once a day, for three consecutive days. Samples were collected after the last EA.

### Immunofluorescence Analyses

After the last EA in the second experiment, the hearts were immobilized with paraformaldehyde, and finally, the brain and colon tissues were collected for use. Phosphate buffer saline (PBS) was used to wash the samples (3 washes, ×10 min/wash), and antigens were extracted with citrate buffer. Samples were then permeabilized and blocked in PBS containing 0.5% Triton X-100 and 5% goat serum (Hyclone, Thermo Fisher Scientific) at room temperature for 1 h. Sections were incubated with primary antibody in blocking buffer overnight at 4°C. After washing, fluorescently labeled secondary antibodies were added to the blocking buffer, and sections were incubated in blocking buffer at 37°C for 1 h in the dark. Then, the samples were washed and counterstained with diamidino-phenylindole (DAPI). Images were scanned with panoramic 250FLASH full scanning microscope (3DHISTECH Ltd.) and then photographed with the software case viewer.

### Western Blot Analysis

After the last EA in the second experiment, colon tissues were collected. Western blot was used to detect the protein expression levels of CRH, CRHR1, CRHR2, and GAPDH in the rat colon. Protein concentrations were determined using a bicinchoninic acid protein assay kit (Beyotime Biotechnology Chain). Protein samples were separated by sodium dodecyl sulfate-polyacrylamide gel electrophoresis (5% concentrated gel and 12% separation gel) and transferred onto a nitrocellulose membrane. The membranes were blocked with PBS containing 0.1% Tween-20 and 5% fat-free milk at room temperature and then incubated with rabbit antibodies against CRH (1:1,000, DF6258, Affinity); CRH-R1 (1:1,000, AF5310, Affinity); CRH-R2 (1:1,000, AF5306, Affinity); and GAPDH (1:1,000, CST). Quantification of total protein was determined relative to GAPDH. Band intensity was densitometrically quantified using ImageJ software (National Institutes of Health).

### Statistics

GraphPad Prism.9.0 software was used for statistical analysis. Data were expressed as means ± SD. Differences between groups were analyzed by one-way analysis of variance (ANOVA), or unpaired *t* test followed by the Tukey method (test) to perform pairwise (multiple) comparisons between group means, and statistical significance was defined at *P* < 0.05.

## RESULTS

### EA Increased Visceral Pain Threshold and Inhibited Intestinal Motility in the IBS Rat Model

**Figure 1.**
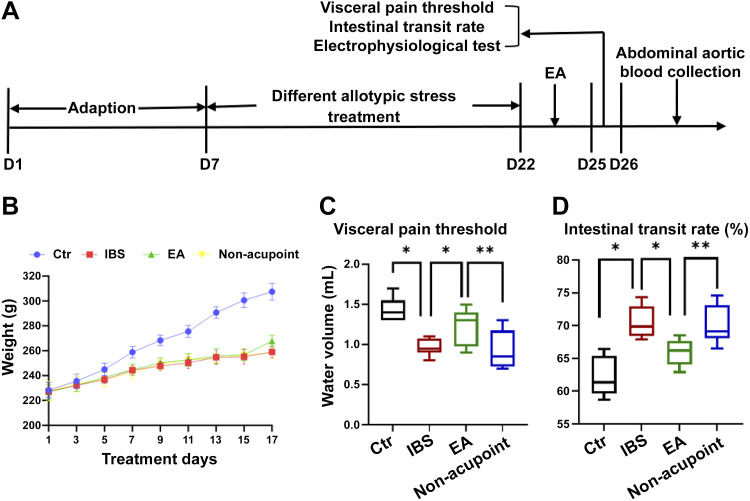
Electroacupuncture (EA) increased visceral pain threshold and inhibited intestinal motility. *A*: experimental process diagram. After 7 days of adaptation, except for the control group rats, the other groups rats were treated with chronic allotype stress for 15 days. Except for the control group and irritable bowel syndrome (IBS) group, the other groups were treated with acupuncture for 3 days. Finally, visceral pain threshold, electrophysiological test, and intestinal propulsion rate were detected, and abdominal aortic blood was collected. *B*: the body weight of rats in each group was recorded every other day for 17 days. *C*: statistical graphics of visceral pain threshold values of each group. *D*: statistical graphics of intestinal propulsion rate values of each group. The data were analyzed using repeated-measures one-way ANOVA and *t* tests. (*n* = 8 rats/group). *P < 0.05 vs. IBS group; **P < 0.05 vs. EA group. Values are presented as means ± SD.

We determined whether EA increased visceral pain threshold and inhibited intestinal motility (Fig. 1*A*). Compared with the control group, the body weight of rats in the IBS group increased slowly. The difference in body weight of rats in the control group was statistically significant compared with the IBS group from the 5th day (*P* < 0.05) and continued until *day 17*; the growth range of body weight of rats in the EA group increased gradually (Fig. 1*B*). Under chronic atypical stress, the visceral pain threshold [*P* < 0.05, *F*(3,28) = 12.55] in the IBS group was clearly decreased and significantly increased in the EA group (Fig. 1*C*). Intestinal propulsion rate [*P* < 0.05, *F*(3,28) = 18.86] in the IBS group was significantly accelerated compared with the control group and significantly decreased in the EA group (Fig. 1*D*).

### EA Decreased the Serum Content of CRH, ACTH, and CORT in the IBS Rat Model

**Figure 2.**
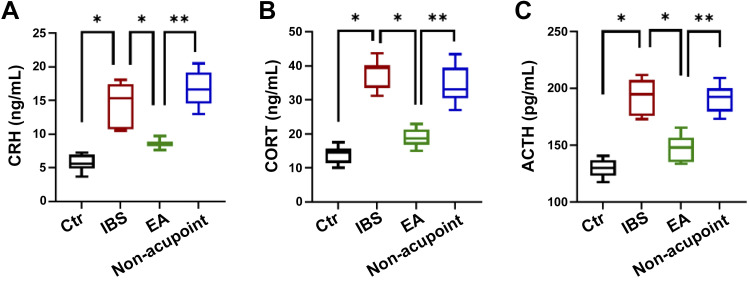
Electroacupuncture (EA) decreased the serum concentrations of corticotropin-releasing hormone (CRH), adrenocorticotropic hormone (ACTH), and corticosterone (CORT) of irritable bowel syndrome (IBS) rats. *A*: statistical graphics of the serum concentration of CRH of each group. *B*: statistical graphics of the serum concentration of CORT of each group. *C*: statistical graphics of the serum concentration of ACTH of each group. The data were analyzed using repeated-measures one-way ANOVA and *t* tests. (*n* = 8 rats/group). *P < 0.05 vs. IBS group; **P < 0.05 vs. EA group. Values are presented as means ± SD.

We determined whether the concentrations of CRH, ACTH, and CORT in the serum of IBS rats under chronic allotypic stress were decreased after EA (Fig. 2, *A–C*). The results showed that the serum concentrations of CRH [*P* < 0.05, *F*(3,28) = 44.09], ACTH [*P* < 0.05, *F*(3,28) = 53.67], and CORT [*P* < 0.05, *F*(3,28) = 70.07] in the IBS rats under chronic atypical stress were significantly increased, whereas the serum contents of CRH, ACTH, and CORT were significantly reduced after EA for 3 days at the rate of 20 min/day. In addition, there was a significant difference between the nonacupoint group and EA group (*P* < 0.05). The results showed that EA could decrease the serum concentrations of CRH, ACTH, and CORT of IBS rats.

### EA Inhibited the Discharge Frequency of PVN Neurons in the IBS Rat Model

**Figure 3.**
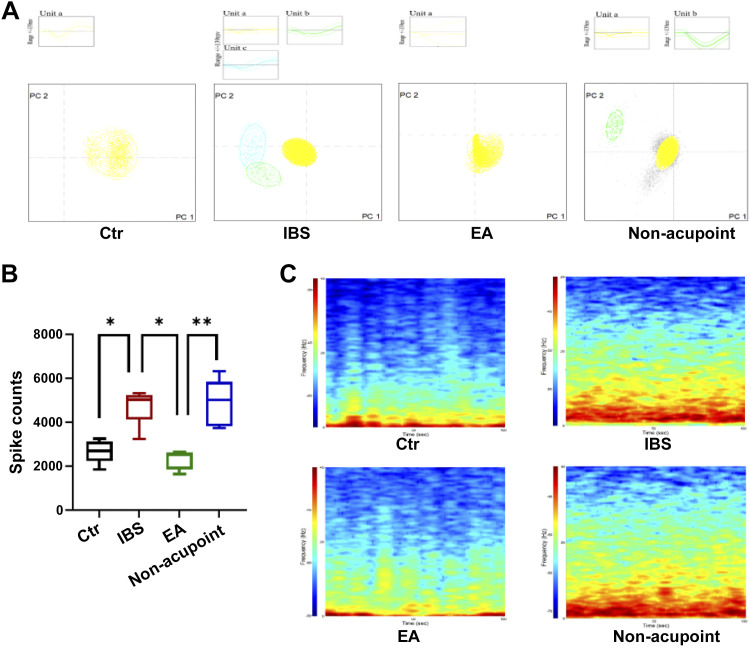
Analysis of paraventricular nucleus (PVN) discharge frequency in each group. *A*: cluster analysis of the discharge frequency of PVN neurons in irritable bowel syndrome (IBS) rats undergoing electroacupuncture (EA). *B*: discharge frequency of the PVN in IBS rats undergoing EA (statistical graphics of rate, time = 300 s). The data were analyzed using repeated-measures one-way ANOVA and *t* tests. (*n* = 5 rats/group). *P < 0.05 vs. IBS group; **P < 0.05 vs. EA group. Values are presented as means ± SD. *C*: real-time spectrum analysis of PVN neurons discharge. The four groups were sequenced as follows: IBS group > nonacupoint > control group > EA group.

We wanted to determine whether EA could inhibit PVN neuronal discharge (Fig. 3). After the PVN neuronal discharges were stable, the Plexon multichannel in vitro recording technology was used to record the PVN neuronal discharges for 5 min. The effective neurons were screened using the Offline Sorter software (Fig. 3*A*). There was one type of PVN neuronal discharge in the control group, three types in the IBS group, one type in the EA group, and two types in the nonacupoint group. The Neuro Explorer software was used to analyze the PVN neuronal discharge [*P* < 0.05, *F*(3,16) = 15.67] and real-time spectrum analysis (Fig. 3, *B* and *C*). The total discharge frequency of the IBS group was significantly higher than that of the control group, and the total discharge frequency of the EA group was significantly lower than that of the IBS group after EA for 3 days at the rate of 20 min/day (Fig. 3*B*). It was confirmed that EA inhibited the discharge frequency of PVN neurons. We observed changes in the spectral characteristics of the signal with time to judge the changes of energy level in a certain period through the changes of color, brightness, and spectral components and to visually express the discharge activity of neurons (Fig. 3*C*). The results showed that the spectrum energy of the local FP in the IBS group was significantly enhanced compared with that in the control group, and the spectrum energy of the local FP in the EA group was significantly lower than that in the IBS group. These results suggest that the spectral energy of PVN may be involved in the mechanism of action of EA on intestinal function in IBS rats.

### EA Regulated the Excitatory and Inhibitory Effects of PVN CRH^+^ Neurons to Affect Intestinal Functional Activity

**Figure 4.**
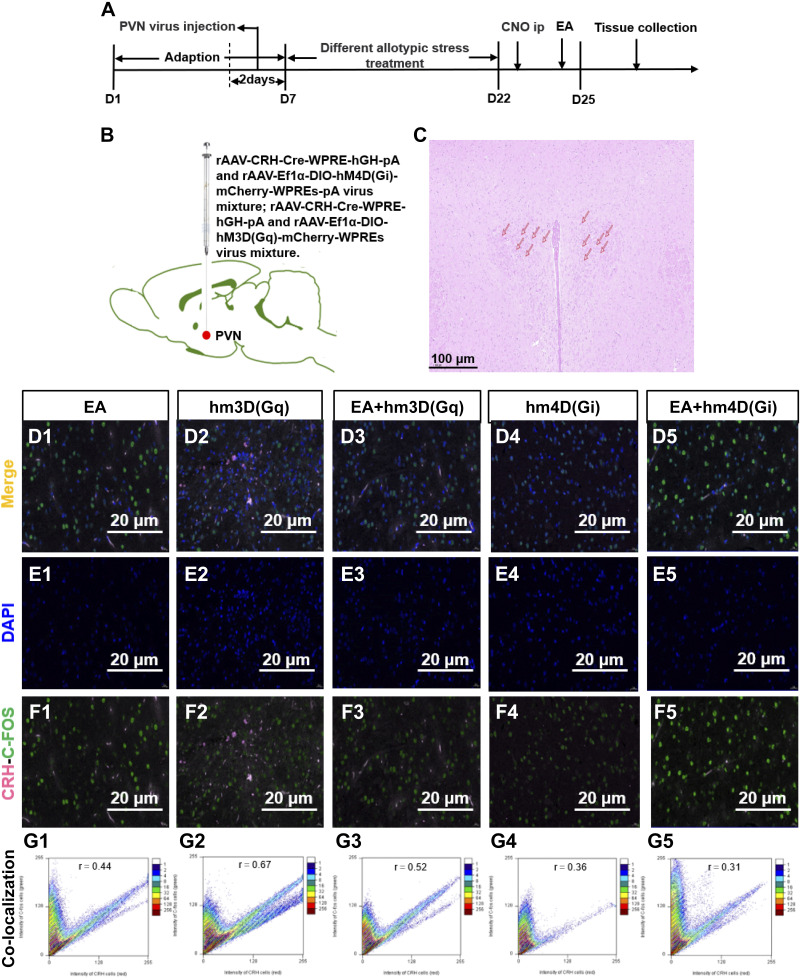
Electroacupuncture (EA) regulated the excitatory and inhibitory effects of paraventricular nucleus (PVN) corticotropin-releasing hormone (CRH) neurons. *A*: experimental process diagram. After 5 days of adaptation, except for the EA group, the PVN of rats in other groups was injected with virus mixture, then each group was treated with chronic allotype stress for 15 days. Except for the hm4D(Gi) group and hm3D(Gq) group rats, the other groups were treated with acupuncture for 3 days, at the same time. Except for the EA group rats, the other groups were intraperitoneally injected with clozapine *N*-oxide (CNO). *B*: virus mixture injection diagram. *C*: PVN (bregma: −1.8 mm, LR: 0.4 mm, and *H*: 8 mm) was accurately localized by hematoxylin-eosin (H&E) (100 µm). *D1*–*F5*: immunofluorescence sections of PVN, DAPI, and CRH-C-FOS of each group (20 µm). *G1*–*G5*: CRH-C-FOS co-labeled scatterplot by Scatter J of each group.

We aimed to confirm whether EA affected IBS by regulating the excitability and inhibition of CRH neurons in PVN (Fig. 4*A*). In the preliminary experiment, kainic acid monohydrate, which can induce nerve injury, was injected into the PVN (bregma: −1.8 mm, LR: 0.4 mm, and *H*: 8 mm), and the cell morphology of PVN was observed through hematoxylin-eosin (H&E) staining (Fig. 4*C*). Compared with the hm3D(Gq) group, the number of CRH-C-FOS co-labeled cells in the EA + hm3D(Gq) group was significantly decreased, and the number of CRH-C-FOS co-labeled cells in the EA + hm4D(Gi) group was significantly decreased. This indicated that EA regulated the excitatory and inhibitory effects of the PVN CRH neurons (Fig. 4, *F1*–*G5*). The number of CRH-C-FOS co-labeled cells in PVN was analyzed by Scatter J, which was a plugin for Image J software. The scatterplot could visually and quantitatively describe the degree of colocalization in the PVN, and its horizontal and vertical axes represent the gray value obtained by each pixel in each channel. The closer the image to the diagonal, the larger the *r* value, and the higher the degree of colocalization (Fig. 4, *G1*–*G5*).

### EA Affected Intestinal Functional Activity by Regulating the Expression of CRHR1 and CRHR2 in the PVN of Experimental Rats

**Figure 5.**
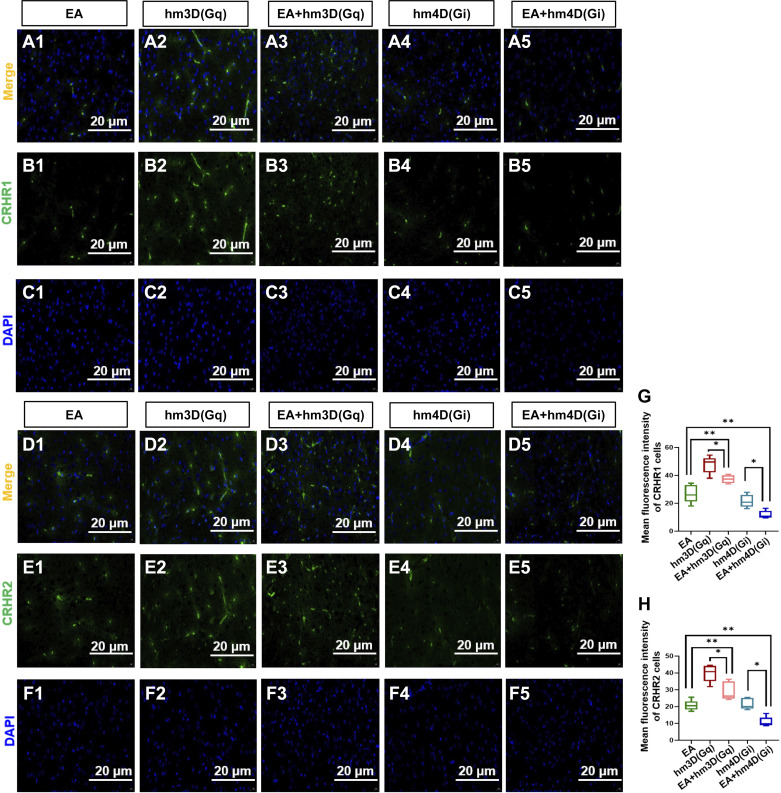
Electroacupuncture (EA) decreased the expression of corticotropin-releasing hormone receptor 1 (CRHR1) and corticotropin-releasing hormone receptor 2 (CRHR2) in the paraventricular nucleus (PVN) of experimental rats. *A1*–*F5*: immunofluorescence sections of PVN, CRHR1, CRHR2, and diamidino-phenyl indole (DAPI) of each group (20 µm). *G* and *H*: mean fluorescence intensity values in statistical graphics of CRHR1 and CRHR2 of each group. The data were analyzed using repeated-measures one-way ANOVA and *t* tests. (*n* = 5 rats/group). *P < 0.05, the hm4D(Gi) group vs. the EA + hm4D(Gi) group and the hm3D(Gq) group vs. the EA + hm3D(Gq); **P < 0.05 vs. EA group. Values are presented as means ± SD.

This study demonstrated whether EA could regulate the expression of CRHR1 and CRHR2 in PVN (Fig. 5). The immunofluorescence sections were analyzed by ImageJ, and the mean fluorescence intensity value was used to analyze the protein expression (Fig. 5, *G* and *H*). Compared with the hm3D(Gq) group, the EA + hm3D(Gq) group showed significantly decreased expression of CRHR1 [*P* < 0.05, *F*(4,20) = 41.60] and CRHR2 [*P* < 0.05, *F*(4,20) = 34.51] in the PVN; there was a significant difference between the hm4D(Gi) group and the EA + hm4D(Gi) group. It was confirmed that EA could regulate the expression of CRHR1 and CRHR2 in the PVN of IBS rats. Compared with the EA group, the EA + hm3D(Gq) group showed significantly increased expression of CRHR1 and CRHR2 in the PVN; there was a significant difference between the EA group and the EA + hm4D(Gi) group. These results suggested that the central CRH nervous system plays an important role in the pathogenesis of IBS.

### EA Affected Intestinal Functional Activity by Reducing the Expression of CRH, CRHR1, and CRHR2 in the Colon

**Figure 6.**
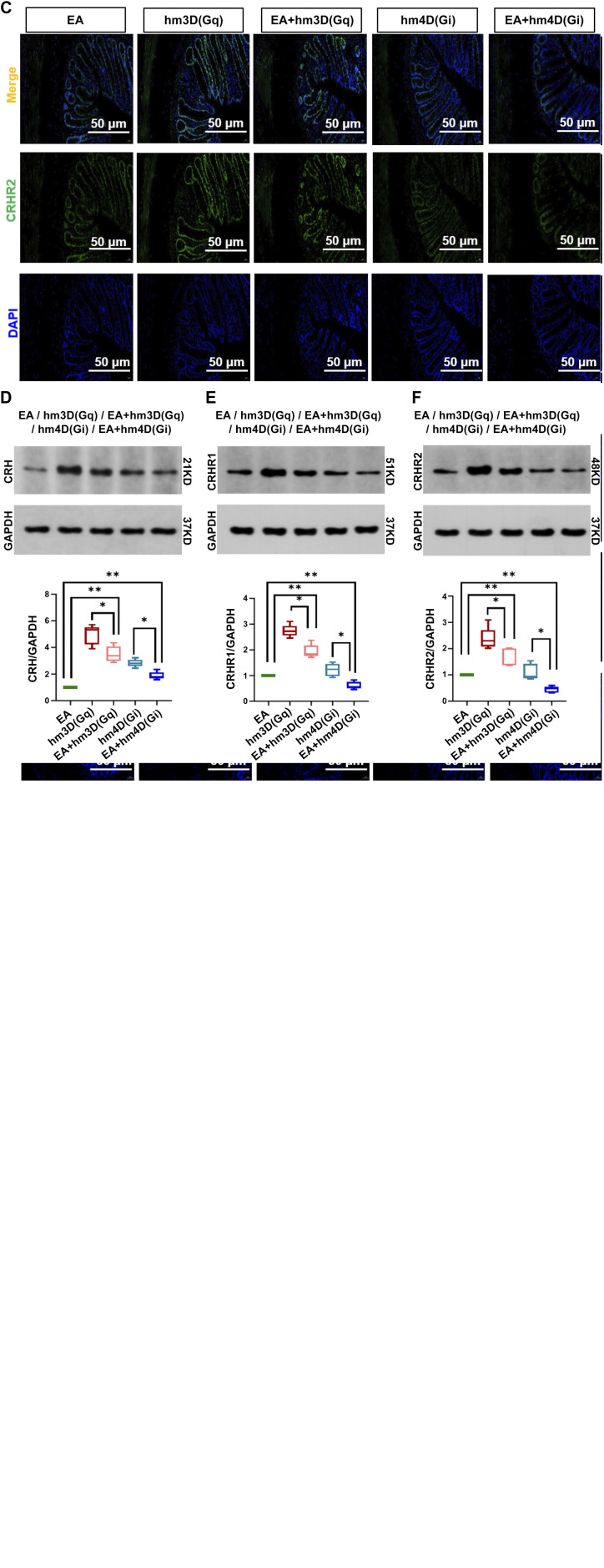
Electroacupuncture (EA) reduced the expression of corticotropin-releasing hormone (CRH), corticotropin-releasing hormone receptor 1 (CRHR1), and corticotropin-releasing hormone receptor 2 (CRHR2) in the colon. *A*–*C*: immunofluorescence sections of CRH, CRHR1, and CRHR2 in the colon from the same site (50 µm). *D*–*F*: Western blot representative stripes CRH, CRHR1, and CRHR2 of the colon and CRH/GAPDH, CRHR1/GAPDH, and CRHR2/GAPDH statistical graphics analysis of each group. The data were analyzed using repeated-measures one-way ANOVA and *t* tests. (*n* = 5 rats/group). *P < 0.05, the hm4D(Gi) group vs. the EA + hm4D(Gi) group and the hm3D(Gq) group vs. the EA + hm3D(Gq); **P < 0.05 vs. EA group. Values are presented as means ± SD.

Next, we determined whether EA could regulate the expressions of CRH, CRHR1, and CRHR2 in the colon (Fig. 6). Compared with the hm3D(Gq) group, the EA + hm3D(Gq) group showed significantly decreased expression of CRH [*P* < 0.05, *F*(4,20) = 5], CRHR1 [*P* < 0.05, *F*(4,20) = 87.47], and CRHR2 [*P* < 0.05, *F*(4,20) = 34.35] in the colon; there was a significant difference between the hm4D(Gi) group and the EA + hm4D(Gi) group. This confirmed that EA could regulate the expression of CRH, CRHR1, and CRHR2 in the colon of IBS rats. Compared with the EA group, the EA + hm3D(Gq) group showed significantly increased expression of CRH, CRHR1, and CRHR2 in the colon, there was a significant difference between the EA group and the EA + hm4D(Gi) group. These results suggested that the CRH nervous system plays an important role in the pathogenesis of IBS.

## DISCUSSION

Our results showed that EA could decrease visceral sensitivity and inhibit intestinal motility in IBS rats; EA could alleviate the increase of the expression of CRH, CORT, and ACTH in the peripheral blood of IBS rats after 15 days of comprehensive stimulation with cold, restraint, hunger, and satiety. EA also inhibited the firing frequency of neurons in PVN, decreased the excitability of CRH neurons in PVN, and reduced the expression of CRHR1 and CRHR2 in PVN. At the same time, the expressions of CRH, CRHR1, and CRHR2 in the colon were decreased. Our results confirmed that EA could regulate intestinal function through the central CRH nervous system and revealed the partial central mechanism of action of EA regulation in IBS rats.

In this study, the cold-restraint stress method was used to replicate the rat model of IBS. Following the improvement of previous research, chronic atypical stress was induced by ice-water swimming, cold restraint, gavage in ice water, abnormal hunger, and satiety (35, 36). The model of this study was established in a natural environment, which was quite similar to the pathogenic causes and mechanisms of patients with clinical IBS and was conducive to our research regarding the mechanism of acupuncture treatment for intestinal function activities in IBS. In this study, chronic allotypic stress stimulation for 15 days in IBS rats could improve visceral sensitivity, reduce visceral pain threshold, promote intestinal motility, shorten stool excretion time, and promote the secretion of stress hormones such as CRH, ACTH, and CORT in peripheral blood. However, it significantly reduced the weight gain of rats. These results were consistent with previous research results (37–39). Chronic allotypic stress stimulation activated the hypothalamo-pituitary adrenal (HPA) axis (40) and promoted the increase of adrenocortical hormone levels, which may be related to the increase and decrease of body weight in rats.

In this study, EA at the ST25, ST37, and LI11 acupoints combined with a pulse electrotherapy instrument was conducive to strengthen the stimulation of acupoints, enhance the sense of deqi, and improve the curative effect (41). According to the meridian theory of traditional Chinese medicine and modern research results, EA at the ST25, ST37, and LI11 acupoints showed improvement in the treatment of gastrointestinal diseases (42, 43). In addition, some researchers have analyzed the advantages of Big Data algorithm, the acupoint selection rules of acupuncture, and the comparison between CCS (Cleveland Constipation Score) symptom score and PAC-QOL (Patient Assessment of Constipation Quality of Life Questionnaire) score, showing that acupuncture at the ST25, ST37, and BL25 acupoints showed particularly significant advantages in the treatment of gastrointestinal diseases (44). Furthermore, acupuncture stimulation signals through unmyelin III (AS), IV (C) afferent fibers, and central nervous system fibers to activate the central nervous nucleus mass to regulate peripheral gastrointestinal hormones, improve gastrointestinal motility, and balance the excitatory and inhibitory effect of intestinal nerves (45). In view of previous research results (31), this study selected the ST25, ST37, BL25, and LI11 acupoints. Combined with a pulse electrotherapy instrument, we verified that acupuncture could regulate the intestinal function activities of the nervous system through the central CRH.

The brain region that is involved in the regulation of the autonomic nervous system, especially the sympathetic nervous system, originates from the PVN (46), and the activation and strengthening of sympathetic nerves are also regulated by the PVN (47), which is an important integrated part of the sympathetic efferent center (48). A recent study found that gut epithelial cells called neuropods were formed at synapses with the vagus nerve, using glutamate as a signaling factor to transmit gut sensory stimuli to the brain stem in milliseconds. This way, the brainstem-vagus link channel was established (49). The PVN is a key structure of the autonomic and neuroendocrine systems to cope with acute and chronic stress (50). Therefore, the PVN was established as a direct signal connection with the vagus nerve. Studies have shown that CRH secreted by neurons of the PVN small-cell population activated the HPA axis, which released ACTH into circulation through the portal system (51, 52). ACTH in turn acts on adrenal cortical cells to promote the secretion of CORT, the major effector hormone of HPA. Therefore, CRH is an important participant in stress response, integrating peripheral and visceral stress-related information to guide neuroendocrine autonomy and behavioral adaptation through the endocrine and neural transmission of PVN (53). In addition, CRH is a key regulatory peptide mediating the hypothalamic-autonomic nervous system pathway to various stress responses and plays an important role in the stress response. Changes in intestinal motility were mainly related to the central role of CRH (8). Mönnikes et al. (11) found that the neuroanatomical site of CRH-mediated changes in intestinal motility was in the PVN. However, the activation of CRH mainly depends on its receptors such as CRHR1, which responded to the released CRH, regulated and coordinated the activation of neurons in the PVN, regulated the output of the HPA axis (54), and was closely related to visceral sensitivity (55). Some studies have suggested that the combination of CRHR2 and its ligand (urocortin2) was involved in the regulation of feeding and intestinal motility (56), and could also induce anxiety (57). Moreover, CRHR2 might have a regulatory function in response to CRHR1 stimulation of the HPA axis or anxiety (58), and CRHR2 played an important regulatory role in the process of visceral pain signaling. Activation of the CRHR2 signaling pathway could alleviate visceral pain sensitivity caused by colon dilatation (59, 60). Thus, in this study, peripheral and central CRH, CRHR1, and CRHR2 were used as indicators for detection. After continuous EA for 3 days, the firing frequency of neurons in PVN and the expression of peripheral and central CRH, CRHR1, and CRHR2 in IBS rats with chronic dysplasia stress were significantly reduced. EA also effectively improved visceral sensitivity and intestinal motility in IBS rats. At the same time, EA could regulate central CRH inhibition or excitability and improve gastrointestinal function by using chemogenetic methods.

Based on the conclusion of this study, we speculated that chronic allotypic stress stimulated the PVN and colon to express a large amount of CRH, which bound to CRHR1 and CRHR2, respectively. The activation of the CRH-CRHR1 signaling pathway decreased the visceral pain threshold, and the CRH-CRHR2 signaling pathway increased the visceral pain threshold. The CRH-CRHR1 signaling pathway played a dominant role in the process of chronic visceral pain in IBS rats. After EA, the CRH-CRHR1 signaling pathway was inhibited, and the CRH-CRHR2 pathway that was the dominant signal pathway was further activated to alleviate the visceral sensitivity of IBS rats. In conclusion, EA could regulate intestinal function through the central CRH nervous system. In addition, chemogenetic methods were used to further reveal the regulatory mechanism of EA in regulating the central part of IBS rats and to provide a scientific basis for the related theories of the meridians, viscera, and brain.

Due to study limitations, the mechanism of the central nervous system related to IBS and acupuncture stimulation is poorly studied. In the first part of the study, we confirmed that EA could improve IBS by observing the results of effect indicators. We confirmed that the improvement of IBS by EA was related to the changes of CRH, CORT, and ACTH in blood. To investigate the CNS mechanism of EA, we applied chemogenetic manipulation of CRH^+^ neurons. In the second part of the study, we found that the central CRH^+^ nervous system plays an important role in the intestinal functional activity of IBS, and EA could regulate intestinal functional activity through the central CRH^+^ nervous system.

## DATA AVAILABILITY

The analyzed datasets generated during the present study are available from the corresponding author upon reasonable request.

## GRANTS

This research was supported by the National Natural Science Foundation of China Youth Program 81904294 and Natural Science Research in Universities of Anhui Province Key projects KJ2020A0423.

## DISCLOSURES

No conflicts of interest, financial or otherwise, are declared by the authors.

## AUTHOR CONTRIBUTIONS

G-Q.Z. and M-Q.Z. conceived and designed research; F.G. and W-H.Y. performed experiments; S-B.W. and Z-B.W. analyzed data; F.G. and W-H.Y. drafted manuscript; F.G. and W-H.Y. edited and revised manuscript; G-Q.Z. and M-Q.Z. approved final version of manuscript.
